# The Effects of Omega-3 Supplementation on Resting Metabolic Rate: A Systematic Review and Meta-Analysis of Clinical Trials

**DOI:** 10.1155/2021/6213035

**Published:** 2021-12-22

**Authors:** Habib Yarizadeh, Bahar Hassani, Saeed Nosratabadi, Hussein Baharlooi, Sara Asadi, Seyed Ahmad Bagherian, Shariful Islam, Kurosh Djafarian, Khadijeh Mirzaei

**Affiliations:** ^1^Students' Scientific Center, Tehran University of Medical Sciences (TUMS), P.O. Box 1417755331, Tehran, Iran; ^2^Department of Community Nutrition, School of Nutritional Sciences and Dietetics, Tehran University of Medical Sciences (TUMS), Tehran, Iran; ^3^Department of Nutrition, Ahvaz Jundishapur University of Medical Sciences, Ahvaz, Iran; ^4^Department of Health Safety and Environment (HSE), Razi Petrochemical Company, Mahshahr, Iran; ^5^Department of Nutrition, Electronic Health and Statistics Surveillance Research Center, Science and Research Branch, Islamic Azad University, Tehran, Iran; ^6^Department of Immunology, School of Medicine, Tehran University of Medical Sciences (TUMS), Tehran, Iran; ^7^Department of Physical Therapy, Faculty of Medical Sciences, Tarbiat Modares University, Tehran, Iran; ^8^The George Institute for Global Health, University of New South Wales, Sydney, New South Wales, Australia; ^9^Clinical Nutrition Department, School of Nutritional Sciences and Dietetics, Tehran University of Medical Sciences (TUMS), Tehran, Iran

## Abstract

**Background:**

It is uncertain if omega-3 polyunsaturated fatty acids are associated with increase in resting metabolic rate (RMR) in adults.

**Objective:**

The aim of the present study was to evaluate the overall effects of omega-3 on RMR.

**Methods:**

Both PubMed and Scopus libraries were searched up to April 2021. Study quality was assessed using the Jadad scale. Random- and fixed-effects models were utilized in order to obtain pooled estimates of omega-3 supplementation impacts on RMR, using weight mean difference (WMD).

**Results:**

Seven studies including a total of 245 participants were included. There was significantly higher FFM-adjusted RMR in the intervention group than the control group (WMD: 26.666 kcal/kg/day, 95% CI: 9.010 to 44.322, *p*=0.003). Study quality showed that four of seven included studies were of high quality. However, there was no significant difference in results in the subgroup analysis according to the quality of studies. Subgroup analyses revealed significant changes for sex (for women: WMD = 151.793 kcal/day, 95% CI = 62.249 to 241.337, *p*=0.001) and BMI (for BMI > 25: WMD = 82.208 kcal/day, 95% CI = 0.937 to 163.480, *p*=0.047). Influence analysis indicated no outlier among inclusions.

**Conclusion:**

The current study depicted that omega-3 polyunsaturated acids can significantly increase RMR in adults. However, further assessments of omega-3 supplementation therapy are critical to monitor its long-term outcomes and potential clinical application.

## 1. Introduction

The global proportion of the aging population is increasing and predicted to reach more than 22% by 2050. Critical changes that appear during aging are increase in fat mass and the reduction of either fat free mass (FFM) or resting metabolic rate [1]. These changes may increase susceptibility to different diseases, particularly diabetes and cardiovascular diseases, which can affect quality of life [2, 3]. As a solution, several studies have suggested increasing the intake of omega-3 polyunsaturated fatty acids (n-3 PUFAs) that exerts beneficial effects by reducing body weight and fat mass through stimulating energy expenditure [4], which may ultimately help elevating the resting metabolic rate (RMR) of individuals.

n-3 PUFAs are natural antioxidants and cofactors for mitochondrial enzymes [5]. Existing evidence has considered n-3 PUFAs as a therapeutic component that influences the metabolic processes of some tissues. For example, it is believed that a higher intake of omega-3 increases the whole-body energy expenditure in the skeletal muscle fibers by changing the activity of membrane-bounded proteins [6–8]. In addition, omega-3 is involved in fat metabolism by changing the expression of proteins such as fatty acid translocase [9]. Considering these properties, n-3 PUFAs may increase the whole-body RMR and promote a shift towards fatty acid oxidation.

However, data provided by human studies have been conflicting. Some studies have indicated that n-3 PUFAs have positive effects on RMR [9, 10]. A study by Christopher et al. indicated that, in a group of healthy young men, supplementation of omega-3s for 12 weeks increased RMR [10]. In contrast, another study revealed that fish oil supplementation did not alter RMR [11]. Therefore, we conducted this systematic review and meta-analysis of the available clinical trials to assess the efficacy of n-3 PUFAs supplementation on RMR in adults.

## 2. Methods

This study was carried out based on the guidelines of the Preferred Reporting Items for Systematic Reviews and Meta-Analysis (PRISMA) statement [12].

### 2.1. Search Strategy

We performed a literature search of the online bibliographic databases (PubMed and Scopus) for relevant publications up to April 2021. In order to find relevant publication, we used the combination of following medical subject headings (MeSH) and non-MeSH keywords: (“Fatty Acids, Unsaturated” OR “Fatty Acids, Omega-3” OR “Fish Oils” OR “Eicosapentaenoic Acid” OR “n-3 Polyunsaturated Fatty Acid “OR “n-3 PUFA (“AND (“Energy Metabolism” OR “Basal Metabolism”) AND (“Clinical Trials as Topic” OR “Clinical Trial” OR “randomized”). Databases were searched by two independent investigators (HY and SA). We also searched for systematic reviews from the abovementioned databases and hand-searched reference lists to identify studies that might have been missed.

### 2.2. Selection of Studies

After removal of duplications, the search results were evaluated by one investigator (SA). Selected studies based on review of the title or abstract were retrieved and reviewed by two investigators. The arguing studies were passed to the third researcher (DJ) for a definite decision of rejection.

### 2.3. Inclusion and Exclusion Criteria for Studies

Eligible publications were included based on the following criteria: (1) investigating population was adults (over 18 years of age); (2) all studies assessed the effects of omega-3 supplementation on RMR or resting energy expenditure (REE); (3) the control group received non-n-3 PUFA (such as olive oil and oleic acid); (4) studies with a design of randomized, controlled clinical trial; (5) human studies; and (6) manuscripts published in English language.

Studies that met the following criteria were excluded: (1) participants younger than 18 years of age; (2) non-RCT designs (observational studies, crossover design studies, letters, review articles, and meta-analysis); (3) studies that did not provide enough data; and (4) studies on specific diseases (such as spinal cord injury (SCI) and acquired immunodeficiency syndrome (AIDS)).

### 2.4. Data Extraction

The study selection and data extraction from each eligible study were conducted independently by two reviewers (HY and SA), and any disagreements were discussed. Data of interest from each individual study were extracted as follows: participant characteristics (first author, year of publication, study population, sample size, age, sex, weight, and BMI) and supplement and placebo details (presence of eicosapentaenoic acid (EPA) and docosahexaenoic acid (DHA), dose, and intervention duration) (Table 1). For three studies which graphically presented their data, the mean and standard deviation were extracted using the GetData Graph Digitizer 2.24 software.

### 2.5. Assessment of Study Quality

Study quality was assessed by a modified Jadad scale [13], in which the total score ranges from 0 to 5 values based on the following criteria: (1) randomization, (2) method of randomization, (3) double blinding, (4) method of double blinding, and (5) report of dropouts and withdrawals. Any discrepancies were resolved by discussion. We defined high-quality publications as those with a Jadad score of 3 or more (Table 1).

### 2.6. Data Synthesis and Statistical Analysis

Data were analyzed using Stata software version 14 (Stata Corp Lp, College Station, TX, USA). Random- and fixed-effects models were utilized in order to obtain pooled estimates of omega-3 supplementation impacts on RMR, using weight mean difference (WMD). Studies that reported two or more interventions of different omega-3 dosages were entered as separate studies. We performed three analyses to compare the effect of omega-3 on (1) RMR; (2) RMR adjusted for body weight; and (3) RMR adjusted for FFM. Within-group mean change was calculated using the difference between baseline and final time point values of RMR (with 95% confidence interval) for either intervention or control groups. Some studies provided a standard error of mean which was used to compute standard deviation according to the formula SD = SEM × square root of *N*. Then, we calculated SD of the mean difference as follows: SD change = square root [(SDbaseline^2^ + SDfinal^2^) − (2 × *R* × SD baseline × SD final)]. Moreover, we determined a correlation coefficient of 0.9 as *R*-value that ranges between 0 and 1. Between-study heterogeneity was examined using the *I*-square (*I*^2^) test. To assess the influence of each study on the overall mean difference, we used a sensitivity analysis by the one-study removal approach. Publication bias was assessed by visual evaluation of the funnel plot and Egger's test. *p* values <0.05 were considered significant.

## 3. Results

### 3.1. Study Selection

**Figure 1:**
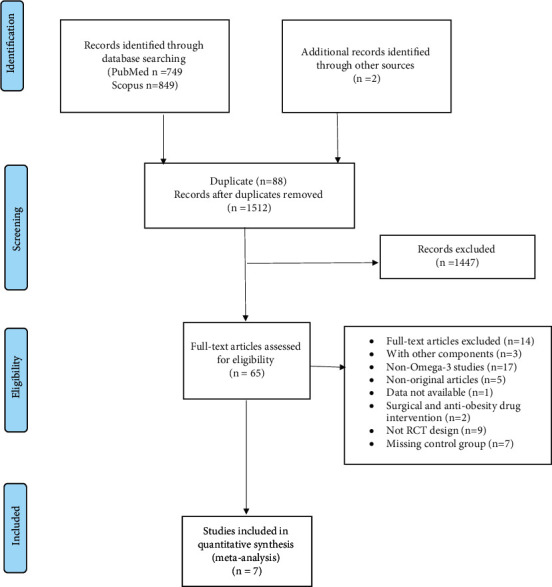
Flow diagram of studies' screening and selection in literature search.

According to the selected search terms, a total of 1512 articles were identified from electronic databases, of which 65 papers were potentially eligible for inclusion after reading the titles and abstracts. Subsequently, 7 studies were found eligible and, therefore, included in the meta-analysis. The remaining articles were excluded due to inaccessibility of the data, additional interventions performed on the participants, missing the control group among others. A flow diagram of the literature search procedure is shown in Figure 1.

### 3.2. Study Characteristics

A total of 245 individuals (with a mean age range of 20 to 68 years) enrolled in the trials, which included 70 men and 131 women; however, gender was not reported for 44 subjects. Of the seven studies in the meta-analysis, two studies were exclusively conducted on women, two exclusively included men, and three studies recruited both sexes. The mean BMI of participants ranged between 20 and 40 kg/m^2^. Most of the participants were healthy adults. The study population comprised of normal weight, overweight, obese, and also, insulin resistance persons. However, a couple of cachectic patients with advanced pancreatic cancer were included. The dose of intervention ranged from 2.2 to 4 grams. The study duration was between 6 and 26 weeks. Additionally, some studies used EPA alone and most of the studies prescribed combination of EPA and DHA (with a ratio of 2 : 1, respectively) (Table 1).

### 3.3. Quality Assessment and Risk of Bias

The quality score of included studies ranged from 2 to 5. Three trials were categorized as low-quality publications (Jadad score <3) and four trials were classified as high quality (Jadad score ≥3). All studies were randomized trials, but four studies did not explain the randomization procedure. Among the seven included studies, three studies were single blind (no study was double blind). All studies reported details concerning with the number of participants that dropped out.

**Figure 2:**
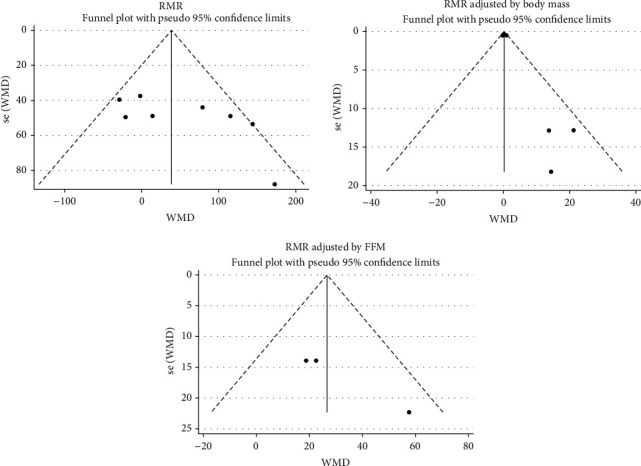
Funnel plot for evaluating publication bias for RMR, RMR adjusted for body mass, and RMR adjusted for FFM.

Visual assessment of the funnel plot denoted no publication bias for RMR, RMR adjusted for body mass, and RMR adjusted for FFM (Figure 2). Accordingly, Egger's test also did not provide evidence of publication bias for RMR (*p*=0.085), RMR adjusted for body mass (Egger's test *p*=0.084), and RMR adjusted for FFM (*p*=0.080).

### 3.4. Outcomes

**Figure 3:**
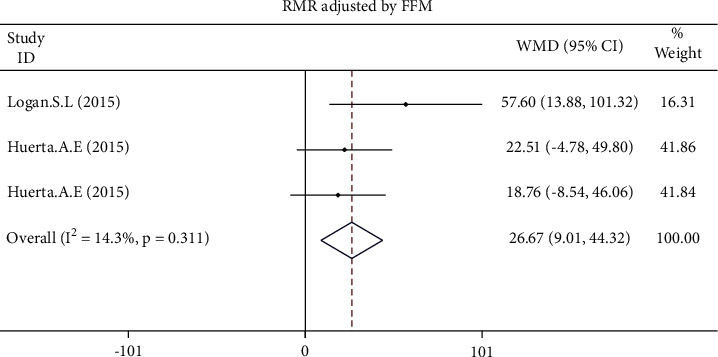
Forest plot presenting weighted mean difference (WMD) and 95% confidence intervals for RMR adjusted for FFM.

**Figure 4:**
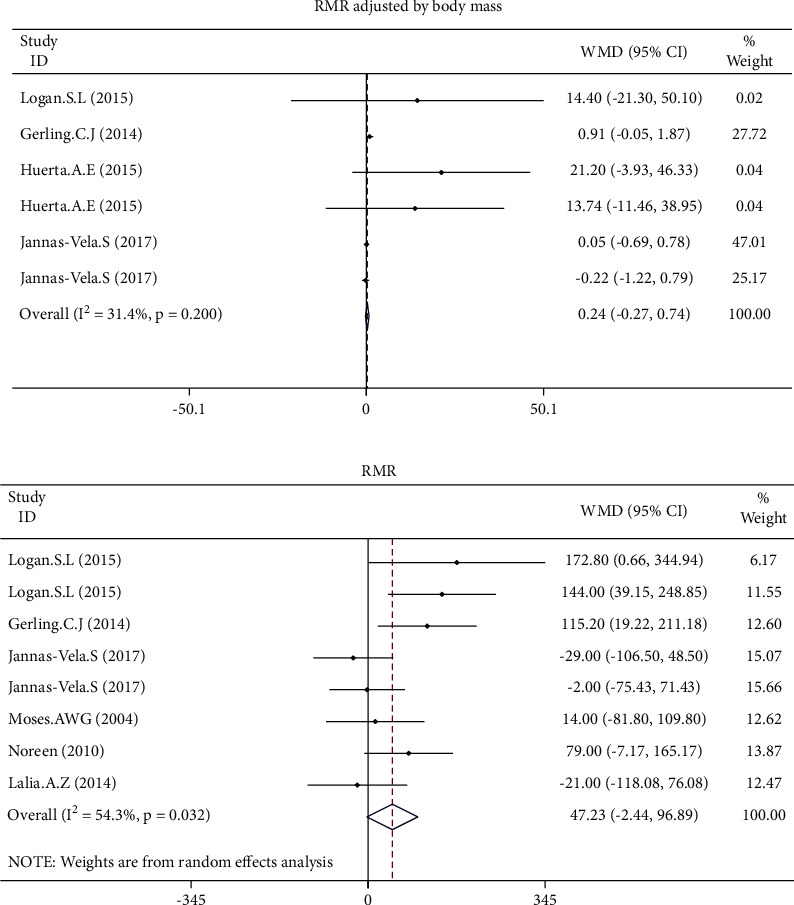
Forest plot presenting weighted mean difference (WMD) and 95% confidence intervals for RMR adjusted for body mass.

**Figure 5:**
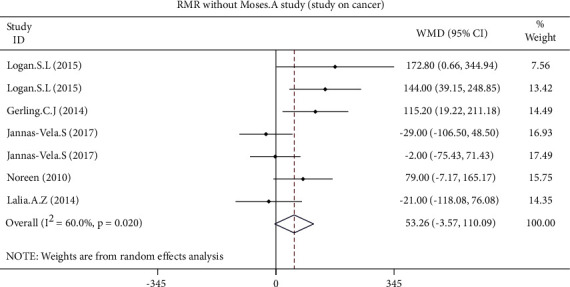
Forest plot presenting weighted mean difference (WMD) and 95% confidence intervals for RMR and intentional elimination of a study carried out by Moses et al. with a population of pancreatic cancer patients.

The pooled effect size of 7 studies demonstrated a significant increase of RMR adjusted for FFM (WMD: 26.666 kcal/kg/day, 95% CI: 9.010 to 44.322, *p*=0.003) following the intervention (Figure 3). In contrast, all changes in RMR (WMD: 47.225 kcal/day, 95% CI: −2.437 to 96.887, *p*=0.062) and RMR adjusted for body mass (WMD: 0.237 kcal/day, 95% CI: −0.268 to 0.741, *p*=0.358) were not statistically significant (Figure 4). The results of the influence analysis did not change the significance level of our findings after the removal of each trial. Furthermore, elimination of a study carried out by Moses et al. in pancreatic cancer patients did not change the statistical outcomes of the study (Figure 5). Finally, the between-study heterogeneity was significant for RMR (I^2^: 54.3%, *p*=0.032).

### 3.5. Subgroup Analysis

To identify the potential sources of heterogeneity, subgroup analysis was conducted according to sex, age, BMI, quality of studies, and dosage of supplement, as well as intervention duration. Significant sources were explored in our meta-analysis including sex (for women: WMD = 151.793 kcal/day, 95% CI = 62.249 to 241.337, *p*=0.001) and BMI (for BMI > 25: WMD = 82.208 kcal/day, 95% CI = 0.937 to 163.480, *p*=0.047).

## 4. Discussion

This is the first systematic review and meta-analysis, to the best of our knowledge, which investigated the effects of omega-3 supplementation on resting metabolic rate in adults. Our results illustrated that the intervention did not significantly change RMR in the study population. Since there was heterogeneity among studies, the subgroup analysis was applied to eliminate heterogeneity. Improvements in subgroup analysis were observed in females and those with a BMI of over 25 kg/m^2^ (overweight and obese individuals). Additionally, significant outcomes were not observed when RMR was adjusted for body mass. Interestingly, omega-3 supplementation led to significantly increased RMR when adjusted for FFM compared to the control group.

Body weight consists of two main parameters: fat-free mass and fat mass. Conflicting studies point to the key role of one of these two parameters as the main determinant of RMR [14]. In contrast, numerous studies have demonstrated that total body weight directly affects RMR [15]. Our data found a significant *p* value for the independency of increased RMR from FFM following omega-3 supplementation. The elevated RMR was significant when we separately analyzed the FFM-adjusted RMR data. Additionally, we found that increase in RMR was no longer statistically significant when RMR was adjusted for body weight after omega-3 intervention. However, studies conducted by Gerling et al. showed that increase in RMR was not affected by body weight [9]. Indeed, RMR changes caused by omega-3 consumption maybe influenced by fat mass, but there was insufficient evidence of fat mass-adjusted RMR data to confirm this hypothesis.

We noticed that omega-3 affects females and people with BMI > 25 more efficiently than males and normal-weight individuals. The molecular mechanism behind the positive effects of omega-3 supplementation on RMR could possibly underlay on the fact that omega-3 increases insulin sensitivity of the tissues without influencing the body weight [4]. In consensus with this explanation, insulin resistance was already reported in omega-3-deficient rats [16] and also in obese individuals who had lower concentration of omega-3 [17]. Furthermore, omega-3 is believed to activate the peroxisome proliferator-activated receptor (PPAR) family [18], and then, the whole complex upregulates the following genes which contribute, particularly, in the metabolism of fatty acids: (1) intra- and extracellular fatty acid transporters (fatty acid-binding protein [19] and fatty acid translocase [20]); (2) ion symporters (such as mitochondrial uncoupling protein 3 which protects the mitochondria from oxidative stress by increasing the proton gradient of the intermembrane space [21, 22]); (3) fatty acid oxidative enzymes [23]; and eventually, (4) a transcriptional coactivator named peroxisome proliferator-activated receptor gamma coactivator 1-alpha as the master regulator of energy metabolism in the mitochondria [24, 25]. Improved glucose tolerance concomitant with higher energy expenditure of the cells generally leads to higher oxygen consumption and metabolic rate.

In line with our findings, previous investigations have also demonstrated that women and overweight people have lower insulin sensitivity [26]. A previous study in healthy females (*n* = 257) has frequently monitored the amount of homeostasis model assessment for insulin resistance (HOMA‐IR) in order to explain insulin resistance. They found HOMA-IR in positive correlation with estradiol and progesterone produced in menstrual cycle. However, there were concerns how accurately HOMA-IR altered the insulin resistance in females [27]. HOMA, together with fat mass and their association with estradiol level [28], was shown to be negatively affected by omega-3 in children [29] as well as adults [30]. According to previous findings, our data suggest that n-3 PUFAs increase RMR level, perhaps through change in HOMA-IR and balanced sensitivity of insulin. However, this needs further evaluation of RMR of n-3 PUFA-consuming subjects with reference to the HOMA, insulin, and glucose level.

The findings should be considered with a few limitations in mind. Firstly, all studies controlled the dietary regiment of participants for three months except for one study. However, attendants in different studies did not use the same dietary intake. Secondly, studies did not use the same equation to calculate the RMR. Additionally, some of the studies had prescribed different amounts of the EPA and DHA. The main strength of this study is that it is, to our knowledge, the first systematic review and meta-analysis which investigated the effects of omega-3 supplementation on RMR.

## 5. Conclusions

Present meta-analysis demonstrated that omega-3 polyunsaturated fatty acids increased the RMR in adult participants, especially in females and those with a BMI of over 25 kg/m^2^ (overweight and obese individuals). Additionally, RMR was shown to be body mass dependent. In contrast, omega-3 supplementation significantly increased RMR when adjusted for FFM compared to the control group. Overall, these data suggest that omega-3 supplementation maybe a healthy approach to increase RMR, consequently preventing from chronic metabolic diseases. However, further long-term studies are needed to evaluate RMR in response to omega-3 with reference to insulin level changes and also metabolism controlling gene expression.

## Figures and Tables

**Table 1:** Demographic characteristics of the included studies.

Study first author (year)	Study population	Gender	Mean age I and P (years)	Mean weight I and P (kg)		Mean BMI I and P	Sample size I and P		Duration (week)	Placebo	Assessment methods of FFM and RMR	Omega-3 daily dose (g)	Jaded score
Moses. AWG (2004)	Cachectic patients with advanced pancreatic cancer	Male and female	68	NR	21–20			7–12	8	n-9 fatty acid (oleic)	RMR: Schofield equations FFM: BIA	2.2 g (EPA)	5
Gerling. C.J (2014)	Healthy active male	Male	22.7–20	82.1–79.0	24			21–9	12	Olive oil	RMR: Péronnet and Massicotte equation FFM: NR	3 g (EPA: 2 and DHA: 1)	2
Lalia. A.Z (2014)	Insulin-resistant humans	Male and female	35.3–32.6	105.3–99.6	35.5–35.2			14–11	26	Softgels oil (oleic)	RMR: NR FFM: DXA	3.9 g (EPA: 2.7 and DHA: 1.2)	4
Noreen (2010)	Healthy adults	Male and female	33–35	71.3–71.1	NR			22–22	6	Safflower oil	RMR: NR FFM: Bod Pod	4 g (EPA:2.7 and DHA: 1.3)	3
Huerta. A.E (2015)	Overweight and obese women during weight loss	Female	38–39	88.4–84.6	Between 27.5 and 40			18–22	10	Sunflower oil	RMR: Weir equation FFM: DXA	1.3 g (EPA)	5
Huerta. A.E (2015)	Overweight and obese women during weight loss	Female	39–38	84.9–83.5	Between 27.5 and 40			17–20	10	*α*-Lipoic acid	RMR: Weir equation FFM: DXA	1.3 g (EPA: 1.3 and *α*-lipoic acid:0.3)	5
Logan. S.L (2015)	Healthy older women	Female	66	72.9–69.1	28–26			12–12	6	Olive oil	RMR: Harris and Benedict equations FFM: BIA	3 (EPA: 2 and DHA:1)	2
Logan. S.L (2015)	Healthy older women	Female	66	72.9–69.1	28–26			12–12	12	Olive oil	RMR: Harris and Benedict equations FFM: BIA	3 (EPA: 2 and DHA: 1)	2
Jannas-Vela. S (2017)	Healthy young man	Male	23–22	77.5–77.8	24			13–13	6	Olive oil	RMR: Péronnet and Massicotte equation FFM: NR	3 (EPA: 2 and DHA: 1)	2
Jannas-Vela. S (2017)	Healthy young man	Male	23–22	77.5–77.8	24			13–13	12	Olive oil	RMR: Péronnet and Massicotte equation FFM: NR	3 (EPA: 2 and DHA: 1)	2

I: intervention; P: placebo; RMR: resting metabolic rate; FFM: fat-free mass; BMI: body mass index; NR: not reported: DXA: dual X-ray absorptiometry; BIA: bioelectrical impedance analyzer.

## Data Availability

No data were used to support this study.
